# Supplementary material for: Neonatal Feeding Practices and SARS-CoV-2 Transmission in Neonates with Perinatal SARS-CoV-2 Exposure: A Systematic Review and Meta-Analysis
Source: J Clin Med. 2025 Jan 6;14(1):280. doi: 10.3390/jcm14010280 (PMC11722486; doi:10.3390/jcm14010280)
Supplement: Supplementary file 1 [file jcm-14-00280-s001.zip › jcm-3382734-supplementary/Supplement S1 material COVID BF_1_2.pdf]

Supplement material

Initial Protocol

Initial protocol: The study initially was designed as a living pragmatic systematic review focused on term and late preterm [≥34 weeks' gestational age (GA)] infants exposed to COVID-19, born to pregnant persons infected within seven days prior delivery or within the next 28 days after delivery. Search was initially conducted using standard Cochrane methods as described in the Cochrane Handbook for Systematic Reviews (Higgins 2019) for articles published from January 1, 2020, to December 31, 2020.

This protocol was published in the Cochrane Library: Babata KL, Yeo KT, Chan CS, Mazzarella K, Adhikari EH, Kong JY, Hascoët J, Brion LP. Feeding strategies to prevent neonatal SARS-CoV-2 infection in term or late preterm babies born to mothers with confirmed COVID-19. Protocol. Cochrane Database of Systematic Reviews 2020, Vol 7 –This protocol was withdrawn in 2021.

It was replaced with:
Babata KL, Yeo KT, Chan CS, Kong JY, Mazzarella K, Sultana R, Hascoët JM, Brion LP. Feeding strategies to prevent neonatal SARS-CoV-2 infection in term or late preterm babies born to mothers with confirmed COVID-19.
https://www.crd.york.ac.uk/prospero/display_record.php?ID=CRD42021268576

Searches and screenings for all articles published from January 1, 2020, to December 31, 2021, using a combination of Medline and Google Search, were conducted by two of the review authors (JMH and LPB). An analysis comparing the original and updated search strategies indicated 94% concurrence in the identification and selection process of the publications in 2020. We included studies that detailed neonatal feeding practices and provided at least one SARS-CoV-2 test result for both the pregnant person and her neonate(s).

The inclusion criteria were: (1) pregnant persons with a positive SARS-CoV-2 PCR test up to 7 days before delivery or within the first 14 days post-delivery for birth hospitalization or up to 28 days for community acquired infection, (2) detailed description of the neonate's feeding method, and (3) availability of the infant's SARS-CoV-2 PCR result within the first 28 days postnatally.

Our search yielded a total of 8390 publications, among which 61 met inclusion criteria, with a total of 152 maternal-infant dyads with maternal SARS-CoV-2 positive PCR at delivery. There was a high risk of publication bias because case reports and case series were the only publications that provided enough details for the detailed analyses planned in the protocol. No randomized trials or case-control studies were available; however, some cohort studies with much larger sample size would have been eligible if entry criteria were modified. The protocol was then modified into the current version detailed in the main manuscript.
