# Supplementary material for: Neonatal Feeding Practices and SARS-CoV-2 Transmission in Neonates with Perinatal SARS-CoV-2 Exposure: A Systematic Review and Meta-Analysis
Source: J Clin Med. 2025 Jan 6;14(1):280. doi: 10.3390/jcm14010280 (PMC11722486; doi:10.3390/jcm14010280)
Supplement: Supplementary file 1 [file jcm-14-00280-s001.zip › jcm-3382734-supplementary/Supplementary S2 Material 01-02-25KB.pdf]

SUPPLEMENTARY FILE S2

I. Search strategy and keywords used

1.1.Initial search strategy June 2020:

The initial comprehensive search strategy was similar to that published in our previous systematic review on delivery room management for the prevention of early neonatal SARS-CoV-2 listed below.[23] Both initial searches were conducted simultaneously.

23. Chan, C.; Kong, J.Y.; Sultana, R.; Mundra, V.; Babata, K.; Mazzarella, K.; Adhikari, E.H.; Yeo, K.T.; Hascoët, J.-M.; Brion, L.P. Optimal Delivery Management for the Prevention of Early Neonatal SARS-CoV-2 Infection: Systematic review and Meta-analysis. Am. J. Perinatol. 2024, 41, 1625–1633. https://doi.org/10.1055/a-2253-5665.

1.2. Updated Search Strategy 2020-2021

A comparison of the initial search strategy and an updated search strategy using PubMed and Google Scholar showed that 94% of the total searched articles were captured using the PubMed and Google updated search approaches. Two reviewers conducted this updated search strategy (LPB and JMH). Updates through PubMed and Google Scholar were subsequently conducted on June 5, 2020, until December 31, 2021.

PubMed Key word search strategy: (*year published) ((covid-19 OR SARS-cov-2 OR coronavirus) AND (neonatal OR newborn OR neonates OR Paediatric OR pediatric OR infants OR children OR pregnancy OR (breast milk) OR (human milk) OR breastfeeding OR (vertical transmission) OR (cord clamping) OR (amniotic fluid))

Google Scholar key word search strategy

((covid-19 OR SARS-cov-2 OR coronavirus) AND (neonatal OR newborn OR neonates OR Paediatric OR pediatric OR infants OR children OR pregnancy OR (breast milk) OR (human milk) OR breastfeeding OR (vertical transmission) OR (cord clamping) OR (amniotic fluid)) NOT (Cohort OR Cohorts) NOT (Commentary OR Commentaries) NOT (Editorial OR Editorials) NOT (analysis OR meta-analysis) NOT (Review OR Reviews) NOT (Vaccine OR Vaccines OR Immunization) NOT (Test OR testing)

The search updated on December 31, 2021 yielded a total of 8390 publications, among which 61 met inclusion criteria, with a total of 152 maternal-infant dyads with maternal SARS-CoV-2 positive PCR at delivery. The list of studies included is available upon request from the corresponding author.

There was a high risk of bias because infants fed with MoM and those not fed with MoM were often not taken from the same population: All infants were from case reports and case series, which were the only publications that provided enough granularity for the detailed analyses planned in the protocol. No randomized trials or case-control studies were available. Preliminary data including case reports and case series analyzed in February 2024 suggested that frequency associated with any MoM was 33/68 or 50.04 % (95% CI 34.09, 65.98) with any MoM and 17/84 or 30.33% (95% CI 19.21,44.34) with no MoM (Table S1).

Table S1. Frequency of neonatal SARS-Cov-2 infection associated with maternal SARS-Cov-2 infection within the last 7 days of pregnancy based on case series and case reports.

Outcomes	Any MOM		No MOM		I^2 (%)	P value
	n	% (95%CI)	n	% (95%CI)		
**Early infection**	16 / 68	31.37 (21.53, 43.24)	15 / 84	27.85 (17.76, 40.82)	4.0	0.67
**Late infection**	17 / 68	32.67 (20.34, 47.97)	2 / 84	18.50 (11.88, 27.64)	0.0	0.07
**All Infection**	33 / 68	50.04 (34.09, 65.98)	17 / 84	30.33 (19.21, 44.34)	37.0	0.07
**Symptomatic infection**	21 / 33	57.02 (40.72, 71.92)	8 / 17	47.57 (29.15, 66.68)	0.0	0.47
**Symptomatic early infection**	6 / 16	42.61 (23.80, 63.83)	6 / 15	43.45 (24.83, 64.12)	0.0	0.96
**Symptomatic late infection**	14 / 17	67.41 (47.11, 82.78)	2 / 2	66.67 (26.81, 91.61)	0.0	0.97

There was no significant difference in frequency of neonatal SARS-CoV-2 infection by type of feeding (Table S2). The frequency of neonatal SARS-CoV-2 infection in these case reports (pooled 34.64%, CI 22.41, 39.30%) was much higher than that in the large US cohort by Hudak 2023 (144/6486, 2.2%).

Table S2. Frequency of neonatal SARS-Cov-2 infection vs type and setting of feeding based on case series and case reports

	Total children	RTP-CR positive	Pooled percentage (95%CI)
**Any MOM**	68	**33**	50.04 (34.09, 65.98)
1. **Breastfeeding without precautions**	27	14	56.88 (35.06, 76.31)
2. **Breastfeeding with precautions**	15	8	52.31 (28.95, 74.70)
3. **Expressed breastmilk**	3	1	40.23 (9.88, 80.51)
4. **Mixed formula feeding (1,2 and/or 3 plus formula)**	9	5	52.78 (27.37, 76.83)
5. **Mixed breastmilk feed (1,2 and/or 3)**	14	5	37.22 (6.72, 82.98)
**No MOM**			
**Formula/donor breastmilk only**	84	17	30.33 (19.21, 44.34)
**Total**	152	50	34.64 (22.41, 49.30)

1.3. Final search 2024

The final search was conducted by LPB on Google Scholar and PubMed Central using the following keywords *(covid-19 OR SARS-cov-2) AND (neonatal OR newborn OR neonates) AND ((breast milk) OR (human milk) OR breastfeeding) AND transmission AND cohort on* March 14, 2024. A preliminary search using same keywords but *randomized trial* instead of *cohort* had revealed no relevant study.

On December 17, 2024 we updated the search on Google Scholar and PubMed Central and added a search of Scopus using the following keywords: *(covid-19 OR SARS-cov-2) AND*

(neonatal OR newborn OR neonates) AND ((breast milk) OR (human milk) OR breastfeeding) AND transmission AND (cohort OR cohorts or randomized clinical trial).

2. Cohorts not included because of overlap with Hudak 2023

Angelidou, USA 2021 [39]
Dumitriu, USA, 2021 [40]
Kunjumon, USA, 2021[41]
Salvatore, USA, 2020 [42]
